# Exploring the impact of early emotional abuse on eating disorder psychopathology: a multiple mediation analysis

**DOI:** 10.1192/j.eurpsy.2025.1389

**Published:** 2025-08-26

**Authors:** E. Barone, M. Carfagno, G. Patriciello, A. M. Monteleone

**Affiliations:** 1 Psychiatry, University of Campania “L. Vanvitelli”, Naples, Italy

## Abstract

**Introduction:**

Childhood emotional abuse (EA) is a well acknowledged risk factor promoting the association between any type of childhood maltreatment and eating disorder (ED) psychopathology.

**Objectives:**

This study investigated the association between EA and ED specific symptoms exploring multiple potential mediators to better understand this complex relationship.

**Methods:**

The study sample included 151 individuals with Anorexia Nervosa (AN), 115 with Bulimia Nervosa (BN), and 108 healthy participants. Before entering treatment programs, participants completed the following questionnaires: the Childhood Trauma Questionnaire, the Toronto Alexithymia Scale, the Behavioral Inhibition System/ Behavioral Approach System, BAS, and the Eating Disorder Inventory-2. A multiple mediation model was run including EA as independent variable, eating symptoms as dependent variables, and ineffectiveness, sensitivity to punishment, alexithymia, and impulsivity as mediators.

**Results:**

In individuals with AN impulsivity emerged as mediator between EA and desire for thinness and bulimic behaviors. In those with BN sensitivity to punishment mediated the relationship between EA and dissatisfaction with body image. In both clinical groups ineffectiveness and difficulty identifying emotions were mediators of the relationship between EA and eating-related symptoms. No mediation effect was observed in healthy controls, although a total effect of EA on dissatisfaction with one’s body was observed.

**Conclusions:**

Impulsivity, sensitivity to punishment, ineffectiveness, and alexithymia may make individuals with childhood EA more vulnerable to ED psychopathology with some differences between AN and BN. Addressing these psychological problems and their connections with early emotional abuse may represent treatment targets for individuals with EDs and a history of childhood trauma.

**Disclosure of Interest:**

None Declared

